# Association between depression and healthcare expenditures among elderly cancer patients

**DOI:** 10.1186/s12888-020-02527-x

**Published:** 2020-03-23

**Authors:** Dian Gu, Robert O. Morgan, Ruosha Li, Ellerie S. Weber, Chan Shen

**Affiliations:** 1 Department of Health Services Research, University of Texas M. D. Anderson Cancer Center, 1400 Pressler St, Unit 1444, Houston, TX TX 77030 USA; 2 Division of Management, Policy and Community Health, University of Texas School of Public Health, Houston, TX USA; 3 Division of Biostatistics, University of Texas School of Public Health, Houston, TX USA; 4 Division of Outcomes Research and Quality, Department of Surgery, College of Medicine, Pennsylvania State University, Hershey, PA USA

**Keywords:** Depression, Healthcare, Expenditures, Elderly, Cancer

## Abstract

**Background:**

Both depression and cancer are economically burdensome. However, how depression affects the healthcare expenditures of elderly cancer patients from payers’ and patients’ perspectives is largely unknown. This study investigated whether depression resulted in higher healthcare expenditures among these patients from both payers’ and patients’ perspectives and identified health service use categories associated with increased expenditures.

**Methods:**

From the Medicare Current Beneficiary Survey (MCBS)-Medicare database, we identified breast, lung and prostate cancer patients aged 65 years and over who were newly diagnosed between 2007 and 2012. Presence of depression was based on self-reports from the surveys. We used generalized linear models (GLM) and two-part models to examine the impact of depression on healthcare expenditures during the first two years of cancer diagnosis controlling for a vast array of covariates. We stratified the analyses of total healthcare expenditures by healthcare services and payers.

**Results:**

Out of the 710 elderly breast, lung and prostate cancer patients in our study cohort, 128 (17.7%) reported depression. Individuals with depression had $11,454 higher total healthcare expenditures, $8213 higher medical provider expenditures and $405 higher other services expenditures compared to their counterparts without depression. Also, they were significantly more likely to have inpatient services. For payers, they incurred $8280 and $1270 higher expenditures from Medicare’s and patients’ perspectives, respectively.

**Conclusions:**

Elderly cancer patients with depression have significantly higher healthcare expenditures from both payers’ and patients’ perspectives and over different expenditure types. More research is needed in depression screening, diagnosis and treatment for this population.

## Background

Studies have shown that the risk of depression is higher for cancer patients than for those with stroke, diabetes and heart disease [1, 2]. While the reported prevalence of depression among cancer patients has varied by study design and definitions of depression, a previous meta-analysis reported a pooled mean prevalence ranging from 8 to 24% [3]. Moreover, cancer patients’ short-term and long-term physical and mental health are negatively impacted by depression comorbidity, as depression has been linked to higher mortality, poorer quality of life, and poorer treatment adherence for cancer patients in general [4–6]. Additionally, as a result of the aging population in the United States and high prevalence of cancer among the elderly, a large portion of cancer patients are 65 and over; it is projected that by 2040 approximately 70% of those diagnosed with cancer will be 65 years or older [7]. Therefore, addressing the mental health needs of elderly cancer patients is vital to improve the wellbeing of both this population and society as a whole.

Aside from its negative health impact as one of the most economically burdensome disorders, depression is usually associated with excess healthcare expenditures. In particular, it has been shown that depression is associated with increased direct healthcare costs for elderly patients with depression [8, 9]. It is plausible that depression can increase the healthcare expenditures since depression can deteriorate the cancer patients’ health and may undermine cancer patients’ treatment adherence [4–6]. However, the magnitude is less known. Only a few studies have examined the higher healthcare expenditures associated with depression among cancer patients [10–13].

A paper focusing on the nonelderly military population showed that military healthcare beneficiaries with both cancer and depression had significantly higher annual healthcare costs compared with those who only had cancer ($16,212 vs $7728) [10]. A recent paper about adult cancer patients aged older than 21 years, showed that compared with those without depression, those with depression had about 32% greater one-year total healthcare expenditures including all third-party payments and out-of-pocket expenditures by patient or family. In particular, depression increased many types of healthcare expenditures, such as total, outpatient, and prescription expenditures for cancer patients. But this paper did not stratify the analyses by payers [11]. A more recent paper examining healthcare charges for cancer patients in the first year after diagnosis from the University of California San Diego Healthcare System found that depressed individuals had 113% higher total annual healthcare charges compared to those without depression [12].

In terms of the impact of depression on elderly cancer patients’ healthcare expenditures, a study examining the association of depression with increased healthcare costs among prostate cancer patients, showed that those with depression had about 30% higher costs compared with those without depression from Medicare’s perspective during the year after cancer diagnosis [13].

Limitations of existing studies include failure to examine overall healthcare expenditures stratified by payers’ and patients’ perspectives, or failure to focus on elderly cancer patients; most did not examine multiple cancer types. Hence, the healthcare expenditures associated with depression in the context of multiple cancer types from the perspective of both payers and patients is not well studied for elderly cancer patients.

## Methods

### Study design

This is a retrospective cohort study that examined the healthcare expenditures associated with depression for elderly cancer patients. In this study, we focused on the three most prevalent cancer types: breast, lung and prostate. We identified cancer diagnosis based on Medicare claims between January 2007 and December 2012; captured depression status based on self-reports from survey data either in the year of cancer diagnosis or the subsequent calendar year; and measured healthcare expenditures in the year of diagnosis and subsequent calendar year after cancer diagnosis. The expenditure measurement period was uniformly at 24 months for all the patients.

### Data source

This study used 2007–2013 Cost and Use files of Medicare Current Beneficiary Survey (MCBS)-Medicare sponsored by the Centers for Medicare & Medicaid Services (CMS). MCBS-Medicare is generated by sampling a nationally representative sample of Medicare beneficiaries, who are surveyed up to three rounds per year for four successive years. The data set contain two types of files that are released annually: Access to Care (MCBS/AC) and Cost and Use (MCBS/CU). The MCBS/CU files were used because they link Medicare claims to survey-reported events. Therefore, the data contains “complete expenditures and source of payment data on all healthcare services,” even if the services are not covered by Medicare. Additionally, the data set contains comprehensive and detailed information on patient demographics, socioeconomic status, healthcare utilization, and self-reported health status and symptoms [14].

### Ascertainment of study cohort

The algorithm to identify cancer patients was based on clinical diagnoses in claims. The beneficiaries were considered to be diagnosed with cancer based on the International Classification of Diseases, Ninth Revision, Clinical Modification (ICD-9-CM) (140–172, 174–208, 225, 227.3 and 227.4) and were required to have at least one inpatient or two outpatient claims or medical provider claims with a cancer diagnosis based on the ICD-9-CM codes. The service date between the two outpatient claims was required to be at least 30 days. Additionally, all patients included in the analytical sample had to be continuously enrolled in Medicare Parts A and B without Medicare Advantage enrollment and not reside in a long-term care facility during the study period so as to ensure the completeness of Medicare claims and prescribed medicine event (PME) files. Patients who were lost to follow-up during the study period were excluded. Newly diagnosed cases were identified by using a 12-month wash-out period.

If clinical diagnosis codes indicated more than one cancer site, this study applied a hierarchical process to assign beneficiaries to the cancer site that is more likely to have been the primary tumor location. For instance, a patient with diagnosis codes for both lung and brain cancer would be assigned to the lung cancer group [15, 16]. Lastly, this study only included beneficiaries belonging to groups of breast, lung and prostate cancer sites with ICD-9-CM codes as 174.x, 162.x, and 185.x, respectively.

### Identification of depression

This study defined the patient as having depressive symptoms via two questions in the survey: [1]. "In the last 12 months, how much of the time did you feel sad, blue or depressed? " [2]. “In the last 12 months, have you had 2 weeks or more when you lost interest or pleasure in the things that you usually cared about or enjoyed?” A patient was considered to have depression if he/she responded “all of the time” or “most of the time” to the first question, and/or answered “yes” to the second question [17, 18]. The combination of these two questions was found to have 91% sensitivity and 86% specificity in detecting depression in cancer and palliative care and hence is a good measure of the presence of depression based on patient self-report [19].

### Dependent variables

The dependent variables included total healthcare expenditures, healthcare expenditures by service types, and payer types. The total healthcare expenditures were combined by MCBS from all payers’ payments and respondents’ out-of-pocket (OOP) payments, and include payments for different services types, including prescribed medicines, dental, home health, hospice, hospital inpatient, skilled nursing facility, medical provider, and hospital outpatient. In addition to total healthcare, we also analyzed subtypes of expenditures by healthcare services and payers. The healthcare services included inpatient (hospital inpatient and skilled nursing facility), hospital outpatient, medical providers, prescribed medicines, and other (i.e., home health, dental, and hospice). The payers included Medicare, other third-parties (i.e., other public [Medicaid, Veterans Affairs Health] Insurance, individually purchased insurance, employer-sponsored insurance, and other payments) and patients’ OOP expenditures. The expenditures were inflated to constant 2017 dollars, adjusting for annual consumer price index for medical care services [20].

The measurement period for expenditures included the year of diagnosis and subsequent follow-up calendar year after cancer diagnosis. While it would have been ideal to measure expenditures in the 12 months following cancer diagnosis, some expenditures include service types and payers that are only reported on an annual basis, such as dental services, OOP costs and other third-party payers.

### Other independent variables

Besides depression status, the selection of the other independent variables was based on the expanded Andersen Behavioral Model [21], which were also identified by self-reports from the survey data. The model is composed of five main constructs: 1) predisposing factors, which included year of cancer diagnosis, gender, age in years at diagnosis and race/ethnicity; 2) enabling factors, which included marital status, educational attainment, poverty status measured as income to percentage of the federal poverty level and supplemental insurance type [16]; 3) need factors, which included cancer site, perceived health status, functional status limitations(the number of activities of daily living (ADLs) with limitations) and the number of comorbid health conditions including heart disease, stroke/brain hemorrhage, hypertension, diabetes mellitus, arthritis, mental disorder other than depression, neurological conditions, and lung disease; 4) personal health practices and use of health services, which included smoking status and body mass index (BMI) [22]; and 5) external environment, measured as metro status in this study. As a result of the flexibility of the model, it can be easily applied to analyze the relationship between various patient characteristics and healthcare expenditures [23, 24]. A valuable list of factors associated with health service utilization for cancer patients were summarized in a recent review paper on Andersen Behavioral Model. (25)The selection of the variables to be included in this study (Table 1) was mainly guided by published studies [23–25] that adopted the Anderson Behavioral Model while taking into consideration data elements available in MCBS-Medicare.

**Table 1:** Characteristics of elderly cancer patients by depression status

Characteristics	Without Depression		With Depression	
	*N*	Wt%	*N*	Wt%
**Total**	582	82.3	128	17.7
**Predisposing**				
**Year of cancer diagnosis**				
2007–2009	317	83.9	63	16.1
2010–2012	265	80.7	65	19.3
**Gender**				
Female	234	79.5	56	20.5
Male	348	84.2	72	15.8
**Age**				
65–74	177	79.4	48	20.6
75 and over	405	84	80	16
**Race/ethnicity**				
Non-Hispanic white	520	82.9	112	17.1
Other	62	76.8	16	23.2
**Enabling**				
**Marital status**				
Married	349	82.8	75	17.2
Other	233	81.2	53	18.8
**Educational attainment**				
Less than high school	114	77.9	34	22.1
High school	213	85.3	39	14.7
Greater than high school	255	81.8	55	18.2
**Poverty status**				
LT 200% FPL	435	84.8	79	15.2
GE 200% FPL	147	74.8	47	25.2
Supplemental insurance**				
Private insurance with Rx	237	83.1	51	16.9
Public insurance with Rx	53	64.2	27	35.8
Medical Insurance only	259	86.1	42	13.9
Other	33	81.9	8	18.1
**Need**				
**Cancer site**				
Breast	208	80.8	44	19.2
Lung	67	80.6	19	19.4
Prostate	307	83.7	64	16.3
Perceived health status***				
Excellent/very good/Good	475	86.4	75	13.6
Fair/poor	107	67.3	53	32.7
Functional status limitation***				
None	426	84.9	78	15.1
≥ 1	156	64.3	50	35.7
Number of comorbid health conditions**				
None or 1	195	88.9	28	11.1
> 1	387	79.1	100	20.9
**Personal health practices and use of health services**				
**Smoking Status**				
Current	38	77.9	11	22.1
Past	319	79.9	76	20.1
Never	225	86.6	41	13.4
**BMI**				
Underweight/normal	206	80.4	54	19.6
Overweight	258	83.7	49	16.3
Obese/morbid obese	118	82.5	25	17.5
**External Environment**				
**Metro status**				
Metropolitan	414	82.5	87	17.5
Non-Metropolitan	168	81.5	41	18.5

***P < .001, **.001 ≤ *P* < .01, *.01 ≤ *P* < .05

Note: *Wt%* Weighted percentage, *LT* less than, *GE* greater than or equal to, *FPL* federal poverty level, *Rx* prescription coverage, *BMI* body mass index

### Statistical analyses

Chi-square test for categorical variables and t-test for continuous variables were used to analyze patient characteristics and healthcare expenditures by patients’ depression status; the tests were weighted using cross-sectional sampling weights [26]. To estimate different types of adjusted additional expenditures associated with depression, generalized linear model (GLM) regressions with gamma distribution and log link, determined by modified park test [27], were used. In the regression analysis, depression status and all other independent variables were included.

In the analyses of healthcare expenditures by service types and payer types, we observed a large number of zeros for some of the expenditure categories such as inpatient and other health services categories of total healthcare expenditures. When the proportion of zero expenditures was non-ignorable, we adopted two-part models [27] with multivariable logistic regressions in the first part and GLMs with gamma distribution and log link in the second part. The first part modeled the probability of utilizing certain services, and adjusted odds ratios (AOR) and 95% confidence intervals (CIs) were provided. The second part estimated the adjusted effect of depression among those who had non-zero expenditures.

All statistical analyses were accounted for the MCBS complex survey design and were performed by using survey sampling and analysis procedures in SAS Enterprise Guide version 6.1 (SAS Institute Inc., Cary, NC) and Stata 14.2 (StataCorp, College Station, TX).

This research article is adapted from a part of Dr. Dian Gu’s dissertation [28].

## Results

The sample included 710 elderly beneficiaries who were newly diagnosed with breast, lung and prostate cancer, among which 128 (17.7%) had depression. We excluded 140 patients due to not having two-year follow-up. Out of these 140 patients, 25 (19.8%) had depression; this percentage is quite close to the 17.7% in the study sample. The MCBS database has up to 3 years of claims data after the initial survey. Therefore, the two-year follow-up requirement essentially excluded patients who received a cancer diagnosis in the last of the three years. Such exclusion is relatively random and we assume that it does not affect the study results. The description of the study sample by depression status is provided in Table 1. Statistically significant differences were found with respect to supplemental insurance, perceived health status, functional status, and number of comorbid health conditions. For example, patients with both public insurance and drug coverage were more likely to report depression (35.8%) compared with those with both private insurance and drug coverage (16.9%), medical insurance only (13.9%) and other supplemental insurance (18.1%).

In Table 2, unadjusted total healthcare expenditures were compared between the patients with depression and those without, presented as total overall expenditures and stratified by service types and payers. Overall, the total healthcare expenditures were significantly higher for patients with depression ($70,918 vs $44,106). In analyses stratified by healthcare service types, patients with depression spent significantly more in medical provider services ($25,052 vs $16,068). Regarding users of other services, those with depression also spent significantly more ($8653 vs $3559). In analysis stratified by payers, patients with depression had significantly more Medicare payments ($48,875 vs $28,856).

**Table 2:** Unadjusted healthcare expenditures by depression status

Healthcare Expenditures	Without Depression (*N* = 582)			With Depression (*N* = 128)		
	*N*	Mean $	SE $	N	Mean $	SE $
**Total healthcare expenditures**						
Overall***	582	44,106	2116	128	70,918	5759
**By service types**						
Medical provider***	582	16,068	934	128	25,052	2609
Hospital outpatient	582	8050	658	128	8006	865
Prescribed medicine	582	7891	485	128	10,188	1242
*In Users*†						
Inpatient	206	28,743	1890	77	35,712	3785
Other*	430	3559	286	97	8653	2152
**By payers**						
Medicare***	582	28,856	1716	128	48,875	4150
Out-of-pocket(patient)	582	6511	291	128	9442	1516
Other third-party payers	582	7950	402	128	11,722	2053
**Medicare healthcare expenditures**						
**By service types**						
Medical Provider**	582	10,832	700	128	15,566	1545
Hospital outpatient	582	5766	501	128	5949	673
*In Users*†						
Inpatient	198	25,658	1850	75	31,072	4458
Prescribed medicine	299	5868	624	69	9659	1969
Other**	103	7077	932	48	12,218	1652
**Out-of-pocket healthcare expenditures**						
**By service types**						
Medical provider**	582	1903	122	128	3028	348
Prescribed medicine	582	1639	98	128	1667	189
Other	582	2067	158	128	4020	1316
*In Users*†						
Inpatient	74	3290	911	27	1685	441
Hospital outpatient	359	823	112	80	659	191

***P < .001, **.001 ≤ *P* < .01, *.01 ≤ P < .05

† Because a large number of patients did not have expenditures in these categories of expenditures, these expenditures were compared only among users (i.e., patients with non-zero expenditures)

Note: *SE* Standard Error

Tables 3 and 4 provides results from adjusted regressions controlling for all the independent variables described in the methods section. Table 3 presents the adjusted total healthcare expenditures and percent change associated with depression from GLM and two-part models, overall and stratified by service types and payers. The results showed that significant differences were found in total healthcare expenditures and also in some total expenditure categories. Patients with depression had $11,454 higher total healthcare expenditures, which corresponded to 34.5% greater total healthcare expenditures. Among different service types, patients with depression had 45.9% higher medical provider expenditures and were significantly more likely to have inpatient services (AOR, 2.94; 95% CI, 1.82–4.74) compared with those without depression. In users of other services, patients with depression had 50.1% greater other services expenditures. In terms of payers, patients with depression not only incurred $8280(43.8%) more expenditures from Medicare’s perspective, but also $1270(32.9%) higher expenditures from patients’ perspective.

**Table 3:** Adjusted effect of depression on total healthcare expenditures, overall and stratified by service types and payers

	AOR [95% CI]	Coefficient (SE)	$ Change [95% CI]	% Change [95% CI]
**Overall**		0.30 (0.09)**	11,454 [4472,19,729]	34.5 [13.5,59.3]
**By service types**				
Medical provider		0.38 (0.1)***	8213 [3477,13,998]	45.9 [19.4,78.1]
Hospital outpatient		−0.79 (0.14)	− 617 [− 2387,1702]	−7.6 [−29.5,21.0]
Prescribed medicine		− 0.07 (0.11)	− 217 [− 819,531]	−6.5 [− 24.6,16.0]
Inpatient‡	2.94 [1.82,4.74]***	0.05 (0.11)	1061 [− 3036,6137]	5.3 [−15.0,30.0]
Other‡	1.05 [0.65,1.69]	0.41 (0.16)*	405 [69,870]	50.1 [8.5107.4]
**By Payers**				
Medicare		0.37 (0.1)***	8280 [3570,13,977]	43.8 [18.87,73.91]
Out-of-pocket(patient)		0.28 (0.13)*	1270 [139,2720]	32.9 [3.60,70.40]
Other		0.23 (0.15)	2613 [− 546,6826]	26.1 [−5.5,68.2]

***P < .001, **.001 ≤ *P* < .01, *.01 ≤ *P* < .05

‡ Because a large number of patients did not have expenditures in these categories of expenditures, two-part models, with logistic regressions in the first part and GLMs with gamma distribution and log link in the second part were used to estimate the adjusted effect of depression

Note: *SE* Standard Error

**Table 4:** Adjusted effect of depression on Medicare and out-of-pocket healthcare expenditures, stratified by service types

	AOR[95% CI]	Coefficient (SE)	$ Change [95% CI]	% Change [95% CI]
**Medicare healthcare expenditures**				
Medical provider		0.31 (0.1)*	4327 [1425,7856]	36 [11.8,65.4]
Hospital outpatient		−0.02 (0.14)	−97 [1180,1327.9]	−2.1 [−25.6,28.8]
Inpatient‡	2.7 [1.59,4.58]***	0.05 (0.12)	922 [3374,6389]	4.8 [−17.6,33.4]
Prescribed medicine‡	0.88 [0.53,1.46]	−0.07 (0.17)	−76 [−387,363]	−6.7 [33.9,31.7]
Other‡	2.55 [1.59,4.09]*	0.39 (0.17)*	870 [113.21921]	47.2 [6.1104.1]
**Out-of-pocket healthcare expenditures**				
Medical provider		0.39 (0.16)*	654 [104,1407]	47.1 [7.5101.3]
Prescribed medicine		−0.02 (0.1)	−10 [−94,93]	−2.3 [−20.9,20.7]
Other		0.43 (0.2)*	465 [21,1130]	53 [2.3128.7]
Inpatient‡	1.71 [0.97,3.01]	−0.54 (0.35)	− 1025 [− 1740,403]	−41.8 [−70.9,16.4]
Hospital outpatient‡	1.05 [0.58,1.92]	− 0.26 (0.22)	− 342 [− 751,296]	−23 [−50.5,19.9]

***P < .001, **.001 ≤ P < .01, *.01 ≤ P < .05

‡ Because a large number of patients did not have expenditures in these categories of expenditures, two-part models, with logistic regressions in the first part and GLMs with gamma distribution and log link in the second part were used to estimate the adjusted effect of depression

Note: SE, Standard Error

Adjusted Medicare and out-of-pocket healthcare expenditures and percent change associated with depression from GLM and two-part models stratified by service types are presented in Table 4. From Medicare’s perspective, among different healthcare services, patients with depression had 36% higher medical provider healthcare expenditures. Patients with depression were highly significantly more likely to use inpatient services (AOR, 2.7; 95% CI, 1.59–4.58) and other services (AOR, 2.55; 95% CI, 1.59–4.09). For patients who used other services, depression was associated with 47.2% greater other services expenditures. From the patients’ perspective, patients with depression had 47.1 and 53% higher medical provider and other healthcare expenditures, respectively.

We did not find sex, race, and education to be strongly associated with expenditures; we did find that patients with more chronic conditions and fair or poor self-reported health status were more likely to have higher expenditures across most health service types.

## Discussion

We found a depression rate of 18% (19% for breast, 19% for lung and 16% for prostate) in our study. These rates fall in the range of 8 to 24%, which was estimated from a meta-analysis of depression prevalence among cancer patients assessed by diagnostic interviews and self-report instruments [3]. Since the prevalence of self-reported depression is high for elderly cancer patients in this study, and depression is often unrecognized for the geriatric cancer population [29, 30], it is essential to improve depression screening and diagnosis for this population. While some instruments such as the Geriatric Depression Scale (GDS) [31] are commonly used to identify depression in the elderly, few studies have assessed their accuracy in the geriatric cancer setting. Considering the complexity and difficulty to identify and detect depression for geriatric cancer populations [32], more research is needed to find or develop accurate, appropriate and validated depression measurement tools.

Our study found that depression was associated with 34.5% greater adjusted total healthcare expenditures, which is consistent with a prior study using 2006–2009 Medical Expenditure Panel Survey data on cancer patients older than 21 years, where the percent increase associated with depression in total expenditures was about 30% [11]. In terms of service subtypes of total healthcare expenditures, depression was associated with greater adjusted medical provider and other services expenditures (45.9 and 50.1%, respectively). Also, depression was associated with higher likelihood of inpatient services use (AOR = 2.94). These findings confirm that depression is correlated with excess healthcare expenditures and utilization for elderly cancer patients, and the higher costs are concentrated on certain services.

When stratified by payers, depression was associated with 43.8% greater adjusted Medicare healthcare expenditures, which is higher than a previous paper (about 30%) about elderly prostate cancer patients from the Medicare perspective [13]. The lower rate identified in that study may be explained by methodology, as the researchers only focused on prostate cancer while the current study included two more cancer types, which may have more influence on the expenditures. When diving deeper into the subtypes, significant findings were found in medical provider, inpatient and other services, suggesting that, as with total healthcare expenditures, the excess is mainly attributable to certain services.

From the patients’ perspective, depression was associated with 32.9% higher OOP expenditures. The OOP expenditures did not include premiums since premiums are separated from actual spending [16].. When expenditures on different service types were analyzed, significant findings were found for medical provider and other services. These findings stress that the excess financial burden of depression is not only placed on the healthcare system but also on the patients themselves, indicating that comorbid depression can aggravate the personal financial burden that cancer patients already face.

Subtype analyses from three aspects (i.e., total [all payers], Medicare and OOP expenditures) all highlighted higher expenditures in the category of medical provider services for elderly cancer patients with depression. In terms of total and Medicare analyses, depression was associated with increased inpatient services use. These results are consistent with previous studies irrespective of cancer diagnosis. For example, two studies of cancer patients using military and Medicare populations demonstrated that cancer patients with depression had more hospitalizations [10, 13]. Also, depression is associated with increased risk of hospitalization in patients with heart failure [33].

It is noteworthy that the estimated expenditures from our study can also contribute to the evaluations of depression-relevant interventions for this population, because the estimates can be applied in cost-effectiveness studies of interventions addressing depression for elderly cancer patients: the reduction of depression related healthcare cost would partially offset the intervention costs.

Since cancer patients with depression incurred substantially higher healthcare utilization and expenditures from payers’ and patients’ perspectives than their counterparts without depression, it is important to manage and treat depression effectively in cancer patients, which can improve health outcomes and potentially reduce healthcare expenditures. It is possible that depression treatment can contribute to higher short-term expenditures (e.g., psychotherapy, psychotropic medications); for long-term outcomes, depression treatment may decrease the expenditures. There has been some research examining how depression treatment affect short-term expenditures but very few studies have examined whether depression treatment has an impact on reducing expenditures in the long-term. Although a study demonstrated that depression treatment (antidepressants, psychotherapy and both) increased healthcare expenditures for elderly breast, colorectal and prostate cancer patients from Medicare’s perspective in the short term but had no effect on long-term expenditures, the study follow-up period of two years after depression diagnosis may not have been long enough [34]. Encouragingly, a recent study demonstrated a relationship between short-term reduction in costs (i.e.the healthcare charges from one year of cancer diagnosis) and increased mental health visits to treat depression for cancer patients with major depressive disorder [35]. Additionally, studies about patients with other co-occurring chronic conditions and depression have shown positive results in reducing costs with depression treatment in both short term and long term. For instance, a study about patients with comorbid conditions and type 2 diabetes mellitus along with depression showed that depression treatment (antidepressants, psychotherapy and both) decreased healthcare expenditures significantly during 12 month period after depression diagnosis [23]. Another study focusing on patients with depression and diabetes showed reduced trends for 5-year mean total medical expenditures when comparing depression collaborative care and usual care [36]. Future research needs to examine whether depression treatment in elderly cancer patients can lower healthcare expenditures, especially in the long run, from payers’ and patients’ perspectives; the depression treatment modalities best suited for this often-vulnerable population need to be elucidated.

This study has many strengths. It makes a significant contribution to the existing literature by estimating the healthcare expenditures associated with depression in the elderly cancer population from payers’ and patients’ perspectives. Also, by examining multiple expenditure categories, our results detail where the excess economic burden of depression originated from in our study cohort. Additionally, because MCBS data links survey to Medicare fee-for-service claims, this study adjusted for a comprehensive list of independent variables, including patient-level health factors that are generally not available in claims data, such as functional status, general health status and personal health practices. Moreover, this study captured complete healthcare expenditures including both Medicare and non-Medicare expenditures.

There are some limitations associated with the data and study design. Firstly, some information such as OOP payments are based on self-report, which may be subject to recall bias. However, MCBS data is an established principle source for assessing OOP cost for Medicare beneficiaries, which is a reliable resource for this study [16]. Moreover, MCBS includes measures to minimize recall bias: for example, the respondents are requested to take their facilitating records of all their healthcare events to the interviews. Secondly, this study is an observational retrospective cohort study, which may have unmeasured confounding factors that cannot be controlled for; thus the results cannot imply causation. Thirdly, the study sample was restricted to fee-for-service Medicare beneficiaries and the results may not be generalizable to other Medicare beneficiaries. Fourthly, the data lacked some information that may also affect healthcare services and expenditures such as severity of cancer [37],severity of comorbid conditions [38] and cognitive status [39]. Fifthly, this study only included breast, lung, and prostate cancer patients, therefore the results may not be generalized to other cancer types.

This study has many important and unprecedented implications. To our best knowledge, it is the first study to provide a national estimate of depression prevalence in elderly patients with breast, lung, and prostate cancer, which are the three most common cancer types in the US, and the excess healthcare cost and utilization burden associated with depression for this population. This study adds to our understanding of the notable economic burden imposed by depression on cancer patients. Additionally, our findings reveal the psychological needs of many elderly cancer patients and their associated higher expenditures; the data may stimulate interest among many stakeholders including policy makers, clinicians, patients and their families. Also, the findings along with some previous study results [40, 41] highlight the importance of effective depression screening, diagnosis, treatment and management. In terms of screening and diagnosis, specific screening/diagnostic criteria need to be implemented with standardized instruments validated in elderly cancer patients with depression. In terms of depression treatment and management, future research needs to examine whether depression treatment in elderly cancer patients can lower healthcare expenditures, especially in the long run, from payers’ and patients’ perspectives. Additionally, as recommended by other studies [41–45], integrated collaborative care treatment models need to be emphasized to monitor and treat depression in this vulnerable population, and other depression treatment modalities suited for this population need to be elucidated.

## Conclusions

In this sample of elderly breast, lung and prostate cancer patients, patients with depression incurred significantly higher healthcare expenditures from payers’ and patients’ perspectives and across different expenditure types. These findings provide compelling evidence for policy makers and clinicians to improve depression screening, diagnosis and treatment in geriatric oncology.

## Data Availability

The data source used for this study is Medicare Current Beneficiary Survey (MCBS) linked with Medicare claims. It is available upon request from the MCBS program.

## Abbreviations

AOR: Adjusted odds ratios
BMI: Body mass index
CIs: Confidence intervals
FPL: Federal poverty level
GE: Greater than or equal to
GLM: Generalized linear models
LT: Less than
MCBS: Medicare Current Beneficiary Survey
Rx: Prescription coverage
SE: Standard Error
Wt%: Weighted percentage
